# Empowering people to help speak up about safety in primary care: Using codesign to involve patients and professionals in developing new interventions for patients with multimorbidity

**DOI:** 10.1111/hex.12648

**Published:** 2017-12-20

**Authors:** Sarah Knowles, Rebecca Hays, Hugo Senra, Peter Bower, Louise Locock, Jo Protheroe, Caroline Sanders, Gavin Daker‐White

**Affiliations:** ^1^ NIHR Collaboration for Leadership in Applied Health Research and Care (CLAHRC) Greater Manchester University of Manchester Manchester UK; ^2^ Division of Population Health, Health Services Research and Primary Care School of Health Sciences Faculty of Biology Medicine and Health NIHR Greater Manchester Primary Care Patient Safety Translational Research Centre (Greater Manchester PSTRC) Manchester Academic Health Science Centre University of Manchester Manchester UK; ^3^ Division of Population Health, Health Services Research and Primary Care School of Health Sciences Faculty of Biology, Medicine and Health NIHR School for Primary Care Research Manchester Academic Health Science Centre University of Manchester Manchester UK; ^4^ Health Services Research Unit University of Aberdeen Aberdeen UK; ^5^ Arthritis Research UK Primary Care Centre Research Institute for Primary Care & Health Sciences Keele University Keele UK

**Keywords:** coproduction, long‐term conditions, patient involvement, patient safety, polypharmacy, primary care

## Abstract

**Background:**

Multimorbidity, defined as the presence of two or more long‐term conditions, is increasingly common in primary care, and patients with multimorbidity may face particular barriers to quality of care and increased safety risks due to the complexity of managing multiple conditions. Consistent with calls to directly involve service users in improving care, we aimed to use design materials to codesign new interventions to improve safety in primary care.

**Design:**

We drew on two established methods—accelerated experience‐based codesign and the future workshop approach. We synthesized design materials based on research into the patient experience of safety and multimorbidity in primary care to enable both patients, service users and carers, and primary health‐care professionals to propose interventions to improve care.

**Results:**

Both patients and professionals prioritized polypharmacy as a threat to safety. Their recommendations for supportive interventions were consistent with Burden of Treatment theory, emphasizing the limited capacity of patients with multimorbidity and the need for services to proactively offer support to reduce the burden of managing complex treatment regimes.

**Discussion & Conclusions:**

The process was feasible and acceptable to participants, who valued the opportunity to jointly propose new interventions. The iterative workshop approach enabled the research team to better explore and refine the suggestions of attendees. Final recommendations included the need for accessible reminders to support medication adherence and medication reviews for particularly vulnerable patients conducted with pharmacists within GP practices.

## 1 INTRODUCTION

Multimorbidity, defined broadly as the coexistence of two or more long‐term health conditions,^1^ is an increasingly “normal” experience for people in later life. As well as placing patients at higher risk of morbidity and mortality, multimorbidity presents challenges for patients in respect of continuity of care,^2^, ^3^ care planning^4^ and self‐management of conditions.^5^ Patients with multimorbidity are therefore at high risk of experiencing disruptions to the quality and safety of their care. It is also increasingly recognized that the burden of treatment itself, for example managing the demands of polypharmacy, may function as an additional barrier to quality of care, safety and good health outcomes.^6^ This has led to calls for existing services to be redesigned to better meet the needs of complex patients living with multimorbidities, with this redesign directly informed by patients and patient experience.^7^

Patient safety in primary care settings is an under‐researched area.^8^ Preliminary work exploring the meaning of “safety” in primary care has uncovered a broad conceptualization that extends to issues such as trust in health services and feeling confident to speak up in consultations with health‐care workers.^9^, ^10^, ^11^ “Safety” is therefore an issue which opens up wider discussions about the physical and social experiences of health care and health‐care systems.^12^ There is an emerging body of research exploring patient involvement in addressing safety in hospital settings,^13^, ^14^ but there has been limited involvement in primary care safety. There is therefore a need to develop interventions that are responsive to the issues specific to primary care and sensitive to patient experiences.

Although guidance such as the Medical Research Council Framework for Complex Interventions encourages the involvement of stakeholders in intervention development, there is a lack of evidence about the best and most efficient methods to achieve this. Conventional qualitative approaches such as interviews and focus groups are effective in capturing patient experience but do not facilitate the explicit involvement of those patients in developing novel interventions. To achieve this, innovative methods based around coproduction approaches may be most useful.^15^ Coproduction approaches are methodologies which explicitly involve patients in design and development. Such approaches are then consistent with NHS calls to involve patients, carers and service users collaboratively in service improvement,^16^ and to work in partnership with patients to improve outcomes.^17^ The success of approaches such as experience‐based codesign (EBCD) have demonstrated the potential for design principles and coproduction methods to enhance patient care in a variety of clinical services including cancer care, emergency medicine and mental health.^18^ In the following study, we aimed to explore whether coproduction methodologies could enhance intervention development and provide a mechanism to translate available evidence into patient‐centred intervention proposals for multimorbidity and safety.

Two methods of participatory design were drawn upon for this study (The EPHESUS study: Empowering People to Help Speak Up about Safety). Firstly, we employed aspects of accelerated experience‐based codesign (AEBCD), a method of rapid EBCD. Specifically, we wished to involve both public contributors and health‐care professionals (HCPs) in the process, in recognition that involving all stakeholders can provide richer insights than involving patients or professionals alone.^19^ We also employed the use of a “trigger film,” a method of distilling patient interviews (in this case from a national narrative interview archive) into a single short film that is intended to act as a “trigger” to stimulate discussion and support identifying improvements. While conventional EBCD requires that individual films are created with the staff and patients at the target site, the trigger film derived from existing interviews enables a more rapid process, and may also be less threatening to professionals than appearing to present critique of “their” service.^20^

Secondly, we drew on the “future workshop” approach, whereby participants attend an initial workshop to critique a current product or service and propose their own ideal solutions and then attend a following session which presents these ideas in prototype form to enable iterative refinement.^21^ Members of the research team (SK, CS, PB) have previously employed this method with patients to propose new designs for mental health technologies.^22^ We aimed to explore whether design materials used in such workshops could be employed to present accessible syntheses of research findings for participants to work with and produce concrete suggestions for improvement. We therefore aimed to synthesize a “persona,” a narrative description of an archetypal patient,^23^ for participants to focus on when proposing improvements to current services.^24^ Consistent with the prototyping employed in the future workshop methodology, we would then use these suggestions to produce “scenarios”^25^, ^26^ which would describe potential interventions in practice, to further explore the acceptability of the suggested solutions and consider their implementation in practice.

To our knowledge, this is the first example of combining the two methods (Table 1), and the first study applying codesign methods to address safety and multimorbidity in primary care. We drew on the iterative prototyping process and synthesized design materials from the future workshop approach. However, it is important to acknowledge that these materials were grounded in genuine experiences (through presenting the trigger film) and engage both patients and professionals together in joint codesign. This approach is consistent with EBCD and AEBCD.

**Table 1:** Elements of each method used in the study

	AEBCD	Future Workshop	EPHESUS
Procedure	Initial separate workshops for patients and professionals, followed by a third joint workshop bringing all participants together	Iterative—initial ideation workshop, followed by critique of “prototypes”	Two workshops with patients and professionals individually, used to create “prototypes” for review in final joint workshop
Materials	“Trigger film” presented at the beginning of the initial workshops to stimulate discussion focused on patient experiences	“Persona” and “Scenario” materials to provide a shared discussion space for identifying solutions	Trigger film used at beginning of individual workshops, followed by persona. Scenario used in third workshop to present “prototypes” for critique

The study therefore had the following aims:

Aim 1: To generate novel interventions, collaboratively with patients and professionals, to address safety issues for patients with multimorbidity in primary care.

Aim 2: To assess the feasibility and acceptability of using participatory design approaches, including persona and scenario materials, to translate research findings through codesign into intervention suggestions.

## 2 METHODS

### 2.1 Procedure

**Figure 1:**
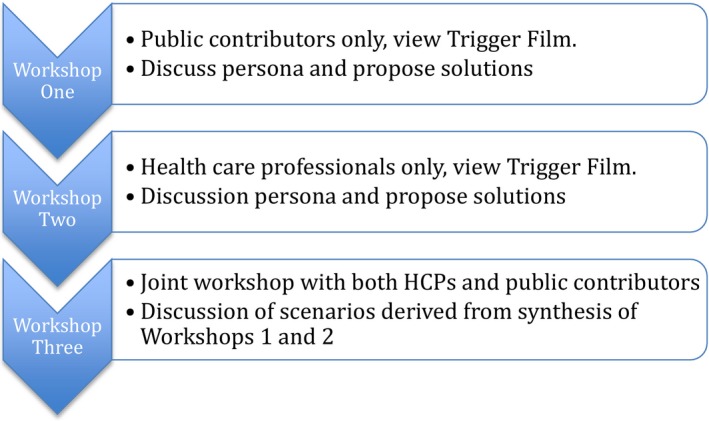
Summary of workshop procedure and content

The procedure is summarized in Table 1. We conducted three workshops between April – July 2016, firstly, separate workshops with HCPs and patients to generate new ideas and then a final joint workshop to review prototypes of those ideas for further refinement. The trigger film was presented to each group in the individual workshops (1 and 2), and then, the persona was provided to focus discussions. The outputs of workshops 1 and 2 were synthesized into prototype scenario interventions for critique in the final joint workshop (Figure 1).

The three researchers facilitating the workshops all had experience of working with patients with multimorbidities and primary care health professionals. Two (SK, RH) had experience of facilitating public involvement activities, and one (SK) had prior experience of facilitating codesign activities and was present for all three workshops.

Ethical approval is not required for involvement activities,^27^ but we sought and received ethical approval to collect and report data on the evaluation of the process (University of Manchester Research Ethics Committee 2 ref 15585, 18 February 2016).

### 2.2 Sample

Nineteen patients, carers and service users (referred to by the summary term “public contributors”) who had previously contributed to public involvement or engagement events within the NIHR Greater Manchester Primary Care Patient Safety Translational Research Centre (Greater Manchester PSTRC) were contacted by email to advertise the workshops, and a summary of the study was presented at a Greater Manchester PSTRC Multimorbidity Research User Group (MRAG) meeting. To contact professionals, we circulated emails and flyers through the PSTRC professional network (a list of primary care health professionals across Greater Manchester who had expressed interest in being involved in research at the centre).

The first workshop, conducted solely with public contributors, was attended by 11 participants who self‐identified as experiencing or caring for someone with multimorbidity (14 participants had expressed interest, and 11 were able to attend on the date). The second workshop was conducted solely with HCPs recruited through Greater Manchester PSTRCPSTRC networks, and was attended by five HCPs—one GP, three pharmacists and one pharmacy dispenser (an additional 3 GPs had expressed interest but were unable to attend on the date). All participants of the previous two workshops were invited to attend the final joint workshop. The workshop was attended by two professionals (one pharmacist, one pharmacy dispenser) and nine public contributors.

### 2.3 Materials

#### 2.3.1 Personas

Personas are intended to be archetypes of the intended users of a service or product, to reflect key characteristics or experiences which should be taken into account when improving a product or service. The persona also provides a shared conceptual space for different participants to focus their suggestions. To effectively present a narrative synthesis of existing patient experience research into safety and multimorbidity in primary care, we drew on the MAXIMUM framework.^8^ This framework provides a taxonomy of events that could lead to harm in primary care, derived from a synthesis of qualitative studies. It also identifies, based on previous research, people with multimorbidities that may be particularly vulnerable to safety failures, for example those on a large number of medications or with low health literacy. We used the taxonomy and risk profile to create the persona of “Elaine” (Appendix S1). The persona was drafted initially by SK, and then reviewed by all members of the study team and a public involvement contributor.

#### 2.3.2 Scenarios

Scenarios are action‐based narratives, in contrast to the character narrative portrayed within a persona. They are therefore more suited than personas to encourage consideration of process and feasibility. In this study, we used the scenarios to prototype example interventions, based on the outputs from workshops 1 and 2 (Appendix S2).

#### 2.3.3 The trigger film

While the persona and scenario were hypothetical creations to aid generation of solutions, the trigger film was included to establish that the issues being explored were grounded in the genuine experiences of real people with multimorbidities. The trigger film was compiled by GDW from an archive of 38 narratives of patient experiences of multimorbidity that was collected for the national Healthtalk patient experiences web site, published in September 2016 (http://healthtalk.org/peoples-experiences/long-term-conditions/living-multiple-health-problems/topics).

### 2.4 Data collection

Workshop discussions were audio‐recorded for review, and researchers present (3 at workshop 1 and 2, 2 at workshop 3) took notes both during the discussions and produced field notes for shared reflection afterwards. We included specific time at the end of each workshop to review the discussions with the participants themselves, to clarify our observations, confirm reflections and ensure that participants felt all key points had been recorded. The multidisciplinary research team (including an academic GP (JP), quality improvement and patient experience researcher (LL), medical sociologists (CS, GDW), clinical psychologist (HS) and health service researchers (SK, RM, PB)) was consulted between each workshop to discuss the suggestions made and agree on content for the “scenarios.”

In workshops 1 and 2, after informal introductions and an overview of the workshop aims, we presented the trigger film and asked participants to reflect on the content. We then presented the persona and asked participants to discuss what safety issues they felt were apparent and which should be prioritized. We then moved onto idea generation, asking participants to propose solutions to the problems they had identified.

In workshop 3, we again completed informal introductions as now HCPs and public contributors were attending together. We then presented the scenarios, and asked participants firstly whether they adequately reflected their suggestions from the previous workshops. Secondly, we asked them to imagine the scenario in practice and whether it would be effective and acceptable for both patients and professionals.

We conducted an evaluation of the process with participants by inviting all attendees to complete an online survey (open for two months following the final workshop), in recognition of calls for more formal evaluation of the process and impact of patient involvement approaches.^28^ Questions from the survey are included in Appendix S3 and asked attendees to reflect on the materials used (persona, scenario, trigger film), the workshop format, and suggest improvements.

### 2.5 Data analysis

We did not preselect an analysis framework for the study outputs, as the workshops were intended to be generative. During the analysis of the discussions of workshops one and two and reflection on the outputs of workshop three, the core constructs from Burden of Treatment Theory^29^ appeared to most effectively capture the study discussions and outputs. We therefore report the outputs of the workshops in reference to this model, but this was applied retrospectively and not used to guide content during the workshops themselves.

## 3 RESULTS

We present results firstly of the workshops themselves, reporting the discussions around safety and suggested interventions (Aim 1). Secondly, we reflect on the process of the workshops themselves and report results of the evaluation (Aim 2).

### 3.1 Aim 1: What interventions to improve safety for people with multimorbidity in primary care were proposed?

The emergent findings, both in terms of the kind of threats to safety that were prioritized and the corresponding interventions required to effectively improve care for patients with multimorbidities, can be understood within Burden of Treatment theory (BoT). BoT focuses on relationships between patients, their social networks and formal services, and considers the capacity of individuals and their networks to perform the work of managing illness with a focus on the need for collective action and compensating for the “burden” of complex treatments themselves. The theory is drawn on here to firstly present the results from the individual workshops (workshop 1, public contributor only, and workshop 2, HCP only, using the “persona” material) and secondly from the joint workshop (workshop 3, using the “scenario” materials.)

#### 3.1.1 Workshops 1 and 2: Outputs

There was consensus in and between the public contributor workshop and the HCP workshop about the risks to safety for patients such as “Elaine,” with medication safety as the primary focus of discussion. The trigger film had focused on medication management (which had emerged as a significant safety issues in the interview); however, patients themselves emphasized that this should not be restrictive and raised issues that had not been covered in the film (such as mental health). Medication management was focused on however in terms of proposing interventions that could address identified problems, which may reflect that it was considered the most “actionable” safety issue.

Given the consensus across workshops 1 and 2 regarding both which threats to safety should be addressed and possible solutions, we present the results together in Table 2.

**Table 2:** Summary of prioritized safety issues and suggestions to address them

Priority risk to safety identified	Suggested solutions
Understanding and management of complex medication schedule, when patient struggles with memory problems and potentially low health literacy	Support to adhere to schedule—reminders, alerts. Explanation by health professionals that is tailored to helping patient understand. Support to share information across different professionals, for example both with GPs and pharmacists
Patient particularly vulnerable—older, multiple conditions requiring different medications, may be suffering from mental health problems such as dementia or depression/anxiety, in dual caring role with husband	Review databases to flag “at risk” individuals such as those over 70 on multiple medications or with comorbidities, and provide those patients with a review to provide additional support
Patient isolated, struggles to organize follow‐up appointments or to know when to contact services. Influence of norms and lack of continuity of care with a trusted professional meaning she worries about “bothering” the health professionals or is unsure who to ask for help	Services must be proactive in offering support Identify whether other people in patient's network (eg family carers) could be included on reminder/appointment notifications Structured assessment to help elicit concerns, particularly around side‐effects of medications and potential adverse interactions

There was some difference in which solutions were discussed more between the two groups, with public contributors focusing on their sphere of influence (the home environment and navigating services) while HCPs focused on theirs (actions during or arranging consultations, communication both with patients and with other professionals). The patient group emphasized the following:

1. The support needed in terms of everyday management at home given the demanding nature of multimorbidity, described by one public contributor as: “…to have multiple conditions and to have to look after someone with multiple conditions is a full‐time job…” This would include support to remember complex medication schedules, but also encompassed the importance of managing side‐effects, particularly the need to prioritize with the patient which conditions should be focused on and which side‐effects were least tolerable.
2. Public contributors also suggested that a way of sharing this information across services (eg from general practice to pharmacy) was necessary, as patients would struggle to remember the information themselves or were required to take responsibility themselves, further adding to the burden of management. One patient commented that “the linkage between them (healthcare professionals) is hugely inefficient and you are running round in circles.”

The professionals also discussed these issues, and public contributors did discuss the need for proactive, supportive and sensitive service provision, but the solutions proposed by the HCPs focused more on the latter. The HCPs spoke particularly about the need for services to explicitly offer support to patients and provide opportunities for them to discuss their concerns, as limited time in consultations and lack of continuity of care meant that the onus was on the patient to initiate such conversations, but they may feel unable to do so.

Consistent with BoT theory, all participants’ discussion therefore encompassed a holistic understanding that recognized Elaine's health and condition management as tied to her personal and social context. The discussions that emerged for both groups of participants can be understood within the concept of “capacity,” which is fundamental to the BoT model. This focuses on the resources available—or lacking—for people managing illness and the demands on their capacity by made complex treatment regimes (such as those faced by patients with multimorbidities). In particular, both patients and HCPs reflected on the limited capacity that “Elaine” had, both in terms of psychological resources (memory problems, possibly low health literacy, anxiety) and physical resources (fatigued, largely housebound, socially isolated) in comparison with an increasingly complex treatment schedule which required considerable organization and also proactive communication with professionals.

However, discussion did not focus solely on “Elaine” herself, but, again consistent with BoT theory, considered the patient within their wider context of relationships both socially, with family members, and with formal health services and professionals. This included recognition of the beliefs or norms about help‐seeking and responsibility that impacted on utilization, for example Elaine's reluctance to “bother” family members and even professionals. In workshop 2, this prompted a sensitive discussion of perceptions of responsibility distributed across HCP networks (“I focus on what I do [checking the medications] but not what I'm not doing [asking the patient about their experience and any side effects]”) and of how interactions with patients could be steered towards limiting conversation that avoided exploring safety risks (“You can ask in a way that they say no everything's ok”).

Following these discussions and prior to workshop 3, the suggestions from workshops 1 and 2 were synthesized into three prototype interventions (Appendix S2):

1. Scenario 1 “In The Practice”: describing a targeted database review, which calls in patients with multimorbidity to discuss their medications, paying specific attention to ensuring health information is understood and patient priorities are sought.
2. Scenario 2 “In The Pharmacy”: describing a pharmacist offering additional support for medication management (such as dosette boxes) by calling in patients who have not collected prescriptions and asking about support available from family or friends.
3. Scenario 3 “In The Home”: describing a “Medication Diary” that can be used to organize medication schedules and provide prompts to attend reviews, and could be shared with health professionals.

#### 3.1.2 Workshop 3: Outputs

Again consistent with BoT theory, discussion of the scenarios focused on the work that was required to effectively manage illness and mitigate against safety risks. Given the limited capacity of people such as “Elaine,” intervention suggestions were critiqued based on how much they compensated for her lack of capacity (eg did they make remembering medication schedules easier, taking into account her memory difficulties?) and how much they drew on the resources of others to help distribute this effort and collectively manage her safety. In relation to the medication diary, it was emphasized that this must go beyond merely monitoring and not appear to be collection of information for the service alone, but should be part of a review process where patients have the opportunity to reflect on the information with a HCP, or should help to overcome capacity issues by providing an accessible means of supporting “Elaine” to adhere to her medication schedule. The idea of an automatic reminder to help patients take medications was therefore reviewed favourably, but using the diary to organize reviews and to share with professionals was considered too burdensome.

One novel focus in workshop 3 (compared to the previous workshops) was on the meaning and shared understanding (between patient and HCP) that was considered vital to underpin any effective intervention, with all participants emphasizing that patients must be convinced of the value of any activities if they are to engage with them and they are to be effective. This is consistent with the importance of “sense‐making” in BoT theory, referring to the work of understanding treatments themselves and communicating needs between actors (patient and professional). Sense‐making is considered an essential component in mobilizing action to enact the work of multimorbidity management. In relation to the enhanced review suggestions, this meant ensuring that reviews were not a “box ticking exercise” and that the HCP conducting the review was both in a position to directly influence treatment (eg changing prescriptions) and able to effectively negotiate these outcomes with the patient. This led to discussion that the pharmacist in Scenario 2 would have to communicate separately with the patient's practice or the onus would be on the patient to request changes, and consequently a pharmacist embedded within practices may be better placed. Participants also emphasized, again consistent with the importance of sense‐making that communication should go beyond checks on understanding of information to communicating the benefits of any changes and being responsive to patient concerns. Both patients and professionals agreed this would require dedicated, protected time to enable issues to be explored.

At the end of workshop 3, the initial ideas represented in the scenario materials had been refined into two specific suggestions:

1. An intervention that provided automatic reminders to support adherence to a medication schedule, and which potentially could also be used to easily communicate the patients’ medication profile to other professionals and capture patient feedback to inform future reviews without providing significant monitoring burden (eg a wearable technology or app).
2. An enhanced review provided by a pharmacist embedded within the patients’ practice (who would therefore be better placed to co‐ordinate with the patients’ GP), ensuring that the goal of the review was communicated clearly to the patient, and the review was collaborative, with the patients’ priorities sought and integrated into the treatment plan.

### 3.2 Aim 2: Evaluation of the feasibility and acceptability of the process to all stakeholders

Participants were positive about the overall experience. Both professionals and patients emphasized the value of the joint workshop, which provided what was considered to be a rare opportunity to openly discuss services with each other.

The online evaluation survey was completed by 7 participants, 4 patients and 3 HCPs. Only one respondent (a patient) attended the joint workshop as well. There were seven respondents, one female, with an average age of 54. Of the four public contributors, two identified as a patient and two as a carer. All were White British except one British Asian (a HCP). The workshop itself was rated as useful (mean rating of 5.9, with 1 being not at all useful and 7 being extremely useful), and the experience was rated as positive (mean rating of 5.3, with 1 being very poor and 7 being very good).

Survey ratings indicated an ambivalent response to the materials, with both the trigger film and the persona/scenario rated as moderately useful (mean rating 3.7 for the persona/scenario and 4.2 for the trigger film, with 1 being not at all useful and 7 extremely useful). However, this reflects that two of the respondents (both patients) did not find the materials useful at all, whereas the others rated them more highly. Telephone interviews with three workshop participants—all patients—added further insights into these matters. While all of those interviewed felt that the trigger film and the persona were useful and informative, difficulties in hearing the film were reported as problematic.

Discussion by patients of their own experiences tended to dominate the discussion in workshops 1 and 3, at the expense of in‐depth discussion of the materials and focusing on proposing ideas to tackle the problems identified. However, responses to the telephone interviews underlined that such exchanges of personal experiences are seen as informative and are highly valued. Other patients however felt that greater balance was needed between discussion of the materials and personal reflections. Two of the patient respondents to the survey commented that “more disciplined” facilitation was needed, commenting that “Elaine’ [the persona] was an opportunity for discussion which was largely lost in participants’ wishes to elaborate on their own” and “Persona should have been more useful, but her problems were lost in too much detail in participants’ personal experiences.” These participants were those who had rated the material as less useful, indicating the problem with these was the facilitation and maintaining focus on them rather than dislike of the materials themselves.

From the research team perspective, the persona was well received. Comments from participants in workshop 1 indicated it was identified with and felt to be representative of genuine experience, with comments including “This is me and my husband on a daily basis” and “This could be my mother‐in‐law this is describing.” The persona provided a useful mechanism for challenging ideas both for facilitators and for patients to explore with each other, commenting “Would that work for Elaine though?” The persona seemed to be particularly powerful for the professional group and prompted a focus on considering the “whole person” experience that the attendees said they may not have considered otherwise. It also appeared to help HCP participants consider different professional perspectives, with one respondent commenting: “It was interesting to discuss the persona with other healthcare professionals, particularly the GP, who approached the persona from a medical perspective, which differed from a pharmacy perspective.”

Although discussion in the joint workshop was focused less on the scenario materials, the scenarios again proved useful for the research team, specifically by providing a means of explicitly checking whether the team's understanding of the suggestions was correct and prompting participants to critique their original ideas. For example, the medicines diary which had been suggested in workshop 1 was reviewed negatively once the scenario was considered, with participants commenting that the diary was an additional burden (as it must be completed and remembered by the patient) and that it was unclear how it could help encourage interactions with HCPs. This led to refinements, considering instead how patients could be helped to remember and report medication use without cognitive cost to themselves, and renewed focus on the responsibility of professionals to be proactive in offering opportunities to discuss medications.

The scenarios also appeared to prompt a focus on “sense‐making” (understanding treatment and sharing this understanding) which was less evident in the first two workshops. It is possible that imagining the interventions in practice helped the respondents to consider motivating factors that would impact on its effectiveness, or this may have emerged due to the joint format with attendees considering whether there is typically shared understanding between patients and professionals.

Although the joint workshop was overwhelmingly attended by patients compared to HCPs and discussion of patients’ own experiences dominated, it was nevertheless evident that the joint format enabled a shared understanding to develop. The pharmacists for example described their responsibilities in checking medicines and the consequences of medicine errors, and patients commented “That's a lot of pressure for you,” indicating empathy and perspective‐sharing with the HCPs. The patient attendees also in some cases referred to their professional experience (as a social worker and as a care home worker), and the HCP attendees referred to their own experience as patients, again indicating that the process enables participants to share perspectives and move beyond restrictive “us and them” conceptualizations.

## 4 DISCUSSION

The present study had two aims—firstly, to codesign new interventions with both patients and professionals to improve safety for people with multimorbidity in primary care, and secondly, to assess the feasibility and acceptability of the codesign process and materials to achieve this. We will firstly discuss how the study met each of these aims, secondly address limitations and finally provide conclusions.

### 4.1 Aim 1: Producing codesigned interventions to improve safety in primary care

The workshop process produced two final intervention suggestions. Common to both is a recognition of the need to compensate for the reduced capacity of vulnerable patients with comorbidities and for organizations to be proactive in providing opportunities to interact with HCPs. Participants furthermore emphasized the importance of providing not only functional mechanisms to achieve this, but ensuring that the purpose and value of such activities is clearly communicated. More broadly, the findings contribute to a growing literature that emphasizes “safety” as not merely the avoidance of error but a need for care which is more holistic and more responsive to patient priorities. Our findings are consistent with research demonstrating that conceptualizations of safety in primary care go beyond technical measures and encompass relational aspects of care that must be negotiated between patient and professional, requiring enhanced communication and sensitivity to the patients’ trust in professionals and treatments.^8^, ^11^

The outputs are also consistent with the finding that patients do not typically have the opportunity to discuss their own needs and preferences regarding medication management,^30^ particularly patients with complex and compounded conditions,^31^ and consequently, mechanisms are required which both provide opportunities for patients to be heard and which support professionals to effectively elicit and incorporate patient views into treatment plans.

The limited capacity of individuals with multimorbidity makes self‐management particularly challenging,^32^ and both patient and professional participants focused on the need for interventions which compensated for, rather than added to, this individual burden. The study outputs were consistent with the Burden of Treatment theory proposed by May and colleagues,^29^ and demonstrate the value of this theory to understanding, and potentially improving, patient safety in primary care. The suggestions are intended to offer prototype ideas for further development, which capture key insights from patients and professionals regarding the need to address treatment burden and enhance communication. The ideas are consistent with the ARIADNE principles for managing multimorbidity in practice^33^ and the core elements (enhancing communication, recognizing patient needs holistically and reducing burden of treatment) could be incorporated into broader interventions such as care planning.

It should be recognized that patients will have different needs and preferences regarding multimorbidity management,^34^ and not all will perceive a “burden.” Participants in the workshops were clear that different needs must be accommodated, and a key recommendation from the workshops was to actively communicate with patients to avoid making assumptions about their capacity or priorities. However, while treatment burden may not be the primary issue in management for all patients in terms of their health outcomes, it may be the priority issue in terms of perceptions of managing safety in treatment, with the workshop outputs supporting the idea that burden of polypharmacy management was considered an especially salient safety threat.

Recent NICE guidance for management of patients with multimorbidity recommends that care is responsive to patient preferences, but the present study enabled us to more specifically explore with both patients and professionals what this could look like in practice. This led to recognition that both structural (dedicated appointments) and relational (sensitive communication, trust) space are essential for such preferences to be genuinely shared. Participatory design approaches therefore appear useful for understanding how recommendations must not only “look” but “feel” in practice.

### 4.2 Aim 2: Assessing the feasibility and acceptability of the codesign methods

The process proved to be feasible and acceptable to all stakeholders (patients, HCPs and researchers themselves), although three issues emerged, firstly regarding recruitment of attendees, secondly the accessibility of the materials and thirdly involving the facilitation burden of the format (these are discussed below in “Limitations”).

Involving both patients and HCPs, consistent with AEBCD, led to a rich understanding of the barriers to improving safety in primary care, with both sets of attendees providing a holistic account that emphasized the interaction between patients’ capacity, the demands of their treatment and the opportunities for intervention in primary care. The final outputs were the product of consensus between all participants.

The structure of the workshops, whereby solutions proposed by each group were used to create prototype interventions for review in the final workshop, drawn from the future workshop approach, demonstrated its value in enabling more iterative design than a single consultation would have allowed. The final workshop and review of the prototypes, in the form of scenarios, enabled us to refine the ideas, challenge our understanding of the suggestions and further synthesize the suggestions into practical concepts.

The “blending” of two methods (AEBCD and future workshops) was successful in enabling us to integrate desired elements from each. Adoption of different methods can be a challenge to fidelity and risk “diluting” key ingredients. We have attempted therefore to clearly describe and justify the elements chosen from each. It is also important to clarify that the workshop process outlined here is not intended to be a substitute for more sustained programmes of participation in improvement initiatives. The workshops are not suggested as a replacement for the need for continued user input to fully develop and evaluate the ideas. The study however demonstrates the feasibility of the methods to provide an early‐stage option for preliminary idea generation and refinement, enabling codesign which could be used to support later coproduction. The use of design materials provided a method for translating research findings into accessible patient experience resources. The study provides further support to calls for greater use of participatory design approaches to support intervention development^21^ and demonstrates the usefulness of such methods to design interventions addressing safety and multimorbidity.

### 4.3 Limitations

There are three key limitations to the study. Firstly, we struggled to recruit as many HCPs as patients. The unbalanced numbers, particularly for the joint workshop, may have contributed to the discussion being dominated by patients discussing their own experiences. However, there was consensus reached around the refinements needed to the prototype interventions, and the HCP‐only workshop outputs were consistent with those from the public contributor workshop. We did consider additional data collection with health professionals through individual surveys/semi‐structured interviews, but felt that this would miss the crucial interactive component of the workshops. Although the number of patients to professionals in the final joint workshop was unbalanced, the outputs suggest that the mix of participants had the desired effect of encouraging multiple perspectives to be considered. A greater number of professional attendees nevertheless may have enabled more divergent views to be captured. We particularly lacked GP attendees however, which may have influenced the final outputs, although patients (and not only pharmacists themselves) suggested the role of community pharmacy in providing support.

Recruitment of the public contributors also had limitations. We are in agreement with arguments that public contributors should not be judged on “representativeness”,^35^ but we do acknowledge that lack of diversity in the attendees means that issues of particular relevance to other groups (such as BAME groups) may have been neglected. This also relates to the second limitation, whereby the use of personas and scenarios to make material more accessible introduced a different barrier to access by limiting attendance to people who can read English. Some patients also expressed difficulties hearing both the video and during the workshops. While design materials can help in making complex concepts more accessible, the accessibility of those materials themselves must be considered, for example whether they are appropriate for people with hearing difficulties (although using a variety of materials, including visual prompts, may overcome this).

The final key challenge was around facilitation, as it was difficult to focus public contributors on the materials rather than on discussion of their own experiences. Discussion of personal experience was not outside the remit of the workshop and we did not wish to exclude such personal reflections, but we had hoped that the workshops would be a space to consider solutions rather than generate new problems. This reflects the challenge of effective facilitation in such settings, where researchers must be sensitive to issues that emerge and provide space for expression, but find a balance between this expression and a focus on the tasks. Involving patients in research and service improvement is a complex task, and as approaches to involving patients also become more complex, it is likely that explicit training and support of researchers to manage involvement activities successfully will be essential.^36^

Finally, there was a limited response to the survey evaluation. More substantial evaluation, for example participant interviews, would be helpful to fully explore participants’ experiences and suggestions for improvement. The positive findings nevertheless provide “proof of concept,” and add to the emerging literature on the value of participatory approaches to improving patient safety, and demonstrate the feasibility and acceptability of integrating the methods.

## 5 CONCLUSIONS

The findings demonstrate that patients and professionals have a shared vision for improving primary care for patients with multimorbidity, perceiving that safety issues may arise from neglecting the burden of treatment on patients with limited resources for self‐management. Focusing on the challenge of polypharmacy, participants emphasized the need for shared effort, with services helping to compensate for the demands of both the patients’ multiple illnesses and the complexity of their treatment. The study demonstrates the value of bringing patients and professionals together to directly contribute to codesign.
