# Supplementary material for: Empowering people to help speak up about safety in primary care: Using codesign to involve patients and professionals in developing new interventions for patients with multimorbidity
Source: Health Expect. 2017 Dec 20;21(2):539–48. doi: 10.1111/hex.12648 (PMC5867321; doi:10.1111/hex.12648)
Supplement: Supplementary file 1 [file HEX-21-539-s001.docx]

**Persona: Elaine**

Elaine is 71 years old and has lived in Bury all her life. She worked part time as a receptionist at the local primary school once her children had grown up, but retired when her husband David had a heart attack and needed help at home. Over the years her own health has gotten worse. She has arthritis and hypothyroidism, and the hypothyroidism gave her hypertension. Her memory isn’t what it used to be, and even little things like going to the corner shop tire her out.

She really liked and trusted her old GP, Dr Baker, but he retired a few years ago. Since then she’s seen a couple of different GPs at the practice. The last GP asked her to come back in a few months after he changed one of her medications, but she can’t remember quite when that was. It might have been before the weather got bad, and she didn’t want to be making journeys to the practice then because the cold made her joints ache and she was frightened she might fall. Her son Anthony offers to drive her but she feels bad asking him to take time off work.

David was meant to go for a review as well but his memory is even worse than hers and neither can remember if his was longer ago or not. The surgery said they’d send a letter but it didn’t come. She can’t remember the new doctor’s name anyway, just that he talked very fast. He seemed nice enough but she just doesn’t feel she’s got the same relationship with him as she had with Dr Baker (

She remembers the doctor telling her to be careful with the different medications and not get them muddled up. The nurse gave her some leaflets about it but her eyesight isn’t very good. She’d ask David to read them but he doesn’t read much, and its full of complicated words, and David says it’s the doctor’s job to make sure the medications aren’t dangerous anyway. The pharmacist told her that some of them need to be taken on an empty stomach but Elaine sometimes forgets and ends up taking them after she’s eaten.

She’s started getting breathless more often, just from walking to the kitchen to make their tea. She doesn’t know if that’s because of the new medication or if it’s something else. David has a pill he takes for his breathlessness, but Elaine doesn’t want to have to take yet another pill as surely taking so many can’t be good for you.
