# Supplementary material for: Empowering people to help speak up about safety in primary care: Using codesign to involve patients and professionals in developing new interventions for patients with multimorbidity
Source: Health Expect. 2017 Dec 20;21(2):539–48. doi: 10.1111/hex.12648 (PMC5867321; doi:10.1111/hex.12648)
Supplement: Supplementary file 2 [file HEX-21-539-s002.docx]

**Scenario 1: In the Practice.**

At the Griffin Practice in Bury, staff perform an enhanced database search that identifies people who are on several medications and may be vulnerable or need extra support, for example those with a mental health problem, those who are carers and those over 70.

The practice nurse, Debbie, has been looking at the search and sees that Elaine hasn’t come in for her most recent follow up. She calls Elaine and invites her to the practice to discuss her medications.

During the appointment, Debbie uses the ‘teach back’ method. Rather than using phrases like “Have you understood?” she says “So that I can be sure I have given you clear instructions, can you describe to me how you would take this pill at home?”

They run through the timing of taking different medications on a chart, and Debbie asks about when and how the pills will be taken. The chart is part of a “My Medications Diary” for Elaine to take away with her. Elaine says she will take her first pill before she makes her breakfast, and Debbie explains she needs to take that pill after she’s eaten a meal. They change the timing on the chart.

Next, Debbie asks “We discussed a lot today. Can you tell me what you found most important?” Elaine says that she worries sometimes about side effects and whether they are things she needs to bother the doctor with. She also worries some side effects will make it difficult for her to do things she needs to at home. She and Debbie draw up a list together of what side effects would worry Debbie, and she would want Elaine to report them, and what side effects matter most to Elaine.

Finally, Debbie explains to Elaine about the services available at her local pharmacy, such as a Medication Usage Review. Debbie flags up on the system a note to appear on Elaine’s next prescription, to remind the pharmacist to ask Elaine about this next time she picks up her medications.

**Scenario 2: In the pharmacy.**

At the Five Oaks Pharmacy in Bury, Steve, one of the pharmacists, is checking their system for uncollected medications. He notices that Elaine hasn’t collected her most recent prescription. He calls Elaine and reminds her about the prescription. He also sees a note from the practice to ask Elaine about a medications usage review.

When Elaine comes to the pharmacy, Steve asks her if she would like to talk through her medications. Elaine says she talked with her nurse about them but it’s still a lot to manage. Even when she knows how to take a particular pill, the writing on the boxes can be so small that she doesn’t know which one is which! Steve suggests he organise the pills into a dosette box to help. He asks if there is anyone at home who could help her do this more regularly, and Elaine mentions her son Anthony comes round every Sunday for lunch. Steve writes down the instructions for matching the pills with the box, for Elaine to give to Anthony.

Steve asks if there was anything that Elaine discussed with the nurse that he could help with. Elaine tells him about their discussion round side effects, and how they rated these, and pulls the notes Debbie made out of her handbag. Steve writes down the telephone number of the pharmacy on the “My Medications Diary”, and tells Elaine she can call up and ask them if she gets worried about any of the side effects she’s experiencing.

**Scenario 3: In the home.**

Elaine has recently had a review with the practice nurse, who has given her some resources to help her manage her medications. Firstly, the nurse wrote everything down that they discussed. This is in a booklet called “My Medication Diary” and includes the timetable for taking the pills, the notes about side effects to report, and now has the pharmacy telephone number from Steve.

Secondly, Elaine has a fridge magnet/sticker that reminds her to check the diary before meal times to see if she needs to take something. Anthony comes round on Sunday for dinner and sees the magnet and asks about it. This reminds Elaine to talk to him about the dosette box. Anthony reads the notes from Steve and puts the box together.

Anthony also sees a space in the booklet which asks if the person would like to nominate someone they know to get reminders about future appointments. Anthony writes down his mobile number, for Elaine to give to the practice next time she has a review. He gets a text message from the practice when she is due another appointment, so he can remind her and offer to drive her to the practice.
