# Supplementary material for: Empowering people to help speak up about safety in primary care: Using codesign to involve patients and professionals in developing new interventions for patients with multimorbidity
Source: Health Expect. 2017 Dec 20;21(2):539–48. doi: 10.1111/hex.12648 (PMC5867321; doi:10.1111/hex.12648)
Supplement: Supplementary file 3 [file HEX-21-539-s003.docx]

1. Are you a GP/Patient or carer?
2. Demographic information: Age/Gender/Ethnicity
3. Have you been involved in research before? (Yes/No)
4. If yes, please give details. (Free text)
5. How useful did you find the workshop with other GPs/other patients carers (delete as appropriate depending on participant)? (1 not at all -7 very)
6. How interesting did you find the workshop? (1-7)
7. How useful did you find the workshop held together with GPs and patients/carers?)? (1-7)
8. How interesting did you find the workshop held together? (1-7)
9. How useful did you find the scenarios and personas for helping identify problems and potential solutions? (1-7 and free text)
10. How useful did you find the trigger film for helping identify problems and potential solutions? (1-7 and free text)
11. How did you find the experience of participating in the workshops? (Free text)
12. Did you experience any problems or barriers? (Free text)
13. What were the best and worst things about how the workshops were organized and run? How might they be done better in the future? (Free text)
14. How do the workshops compare to other involvement activities, if you have previously been involved in research? (Free text)
